# 2D-DIGE proteome analysis on the platelet proteins of patients with major depression

**DOI:** 10.1186/1477-5956-12-1

**Published:** 2014-01-03

**Authors:** Tiao-Lai Huang, Mei-Lan Sung, Tai-Yuan Chen

**Affiliations:** 1 Department of Psychiatry, Kaohsiung Chang Gung Memorial Hospital and Chang Gung University College of Medicine, 123 Ta-Pei Road, Niao-Sung, Kaohsiung 833, Taiwan; 2 Department of Food Science, Center of Excellence for the Oceans, National Taiwan Ocean University, 2 Pei-Ning Road, Keelung 202, Taiwan

**Keywords:** 2D-DIGE, Western blot, Major depression, Platelet, Fibrinogen

## Abstract

**Introduction:**

Platelet activation is related to the psychopathology of major depression. We attempted to search and identify protein biomarkers from the platelets of patients with major depression. High resolution two-dimensional Differential Gel Electrophoresis (2D-DIGE), the matrix-assisted laser desorption/ionization time-of-flight mass spectrometry (MALDI-TOF MS), Western blot, and bioinformatic tools were applied to examine the platelet proteins of 10 patients with major depression and 10 healthy controls.

**Results:**

The levels of 8 proteins were significantly different between the patients with major depression in the acute phase and healthy controls. The levels of protein disulfide-isomerase A3 (PDIA3) and F-actin-capping protein subunit beta (CAPZB) were higher in patients with major depression than in healthy controls. The levels of fibrinogen beta chain (FIBB), fibrinogen gamma chain (FIBG), retinoic acid receptor beta (RARB), glutathione peroxidase 1 (GPX1), SH3 domain-containing protein 19 (SH319), and T-complex protein 1 subunit beta (TCPB) were lower in patients with major depression than in healthy controls.

**Conclusions:**

Platelet provided valuable information about the pathways and processes of inflammation/immunity, oxidative stress, and neurogenesis, related to major depression.

## Background

Several theories have been proposed regarding the pathogenesis of major depression, including the monoamine theory of norepinephrine and serotonin (5-HT), systemic immune activation, and neuroplasticity regulation of brain-derived neurotrophic factors (BDNF). In addition, the role of tissue-type plasminogen activators has also been linked to BDNF from the viewpoint of platelets [1-7], which are related to BDNF and serotonin release [8,9]. Furthermore, many papers have discussed the role of platelet activation in the psychopathology of major depression [10-14].

In our previous study, we developed a technique combining acid hydrolysis with matrix-assisted laser desorption/ionization time-of-flight mass spectrometry (MALDI-TOF MS) for the rapid study of changes in the serum levels of positive and negative acute phase protein biomarkers in patients with major depression. The results suggested that patients with major depression had an increased level of fibrinogen or a decreased level of transferrin [15]. These molecules were related to the platelets.

MALDI-TOF mass spectrometers are extremely sensitive instruments and have now been accepted as a major analytical tool for the detection, identification, and characterization of large biomolecules [16-18]. Two-dimensional differential gel electrophoresis (2D-DIGE) using CyDye DIGE Fluor minimal dyes has also been established and used for studying the differential expression of proteins [19,20].

A recent review has deeply elucidated that a bidirectional relationship between major depression and cardiovascular disorder (CVD), associated with shared inflammatory and oxidative and nitrosative stress (IO&NS) pathways [21]. Lines of evidence showed that IFNγ, a Th-1-like cytokine which production is increased in major depression [22], is a significant factor in atherogenesis and coronary artery disease (CAD) [23]. The other pro-inflammatory cytokines that are increased in depression and that plays a role in CAD are IL-12 [24], IL-1 type, IL-8 [25], IL-2, IL-6 and TNF-α [26,27]. The patients with major depression and animal models of depression were accompanied by induction of IO&NS pathways. The radical oxygen (ROS) and nitrogen species (RNS) production, demonstrated by increase levels of peroxide [28], nitric oxide (NO) productions [29,30] and xanthine oxidase (XO) activity [31] that may react with fatty acid, proteins and DNA, and caused damage.

Recent studies demonstrate that BDNF [32,33], neurotrophin-3 (NT-3) [34], glial cell line-derived neurotrophic factor (GDNF) [35], fibroblast growth factor-2 (FGF-2) [36] and nerve growth factor (NGF) [37] influence adult hippocampal neurogenesis and regulate synaptic plasticity in neuronal networks involved in major depression. Furthermore, BDNF signaling was found to be associated with phosphoinositol dependent kinase 3, Akt and glycogen synthase kinase (GSK3) pathways [38-40].

It remains unclear that differences of the platelet protein between patients with major depression and healthy controls. Therefore, in the present study, we attempted to use the proteomic methods of 2D-DIGE, MALDI-TOF MS, Western blot and bioinformatic tools to compare the platelet protein profile between patients with major depression and healthy controls.

## Results

This study recruited 10 patients (mean age = 37.2 (+/- 9.3) years; mean body mass index = 24.2 (7.0) kg/m^2^; female/male = 6:4) and 10 healthy controls (mean age = 33.1(+/- 6.3) years; mean body mass index = 22.8 (2.7) kg/m^2^; female/male = 6:4). The relative increase of at least 1.5-fold in the abundance of protein spots in normal and patient samples is shown in Table 1. The items include mascot score, protein ID, accession number, protein name, Mr/PI and sequence coverage. Moreover, all matched and unmatched masses, the matched peptide sequences information, enzyme cleavage specificity, any modification and deviations were provided in the Additional file 1: Table S1.

**Table 1:** Relative changes in the abundance of different protein spots with at least 1.5-fold in control and patient samples

**Spot no.**	**Mascot score**	**ID**	**Accession no.**	**Protein name**	**Mr/PI**	**SC**	**Description**
H3(872)	88	FIBB	P02675	Fibrinogen beta chain	55/8.5	35	N>P
H4(867)	62	FIBB	P02675	Fibrinogen beta chain	55/8.5	26	N>P
H6(1026)	72	FIBB	P02675	Fibrinogen beta chain	55/8.5	29	N>P
H7(1023)	114	TCPB	P78371	T-complex protein 1 subunit beta	57/6.0	41	N>P
G11(1014)	57	FIBG	P02679	Fibrinogen gamma chain	51/5.3	18	N>P
G13(1534)	63	SH319	Q5HYK7	SH3 domain-containing protein 19	86/8.5	11	N>P
G15(812)	61	RARB	P10826	Retinoic acid receptor beta	50/8.0	11	N>P
H16(1572)	57	GPX1	P07203	Glutathione peroxidase 1	21/6.1	23	N>P
G1(791)	86	PDIA3	P30101	Protein disulfdte-isomerase A3	56/5.9	25	P>N
H2(786)	94	PDIA3	P30101	Protein disulfide-isomerase A3	56/5.9	30	P>N
G14(1317)	70	CAPZB	P47756	F-actin-capping protein subunit beta	31/5.3	26	P>N

SC: sequence coverage.

**Figure 1:**
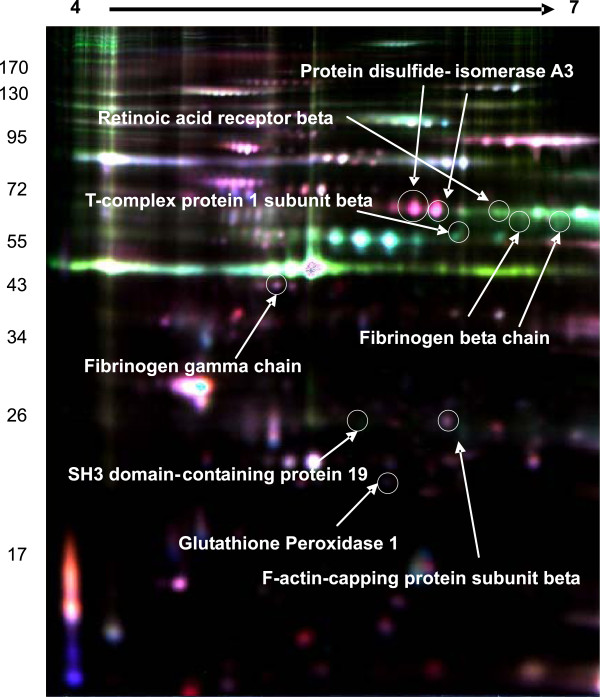
**Representative overlapping 2D-DIGE expression maps of platelet proteins labeled with fluorescent dyes (Cy2, Cy3, and Cy5).** The 2D-DIGE overlay image of protein spots compares a healthy control sample labeled with Cy3 (green) to a patient sample labeled with Cy5 (red) and an internal standard sample common to all gels labeled with Cy2 (blue). This image is representative of 1of the 10 gels analyzed.

Eight proteins were identified among the patients with major depression in the acute phase and healthy controls (Figure 1). The levels of protein disulfide-isomerase A3 (PDIA3) and F-actin-capping protein subunit beta (CAPZB) were higher in patients with major depression than in healthy controls. The levels of 6 proteins, fibrinogen beta chain (FIBB), fibrinogen gamma chain (FIBG), retinoic acid receptor beta (RARB), glutathione peroxidase 1 (GPX1), SH3 domain-containing protein 19 (SH319), and T-complex protein 1 subunit beta (TCPB) were lower in patients with major depression than in healthy controls.

**Figure 2:**
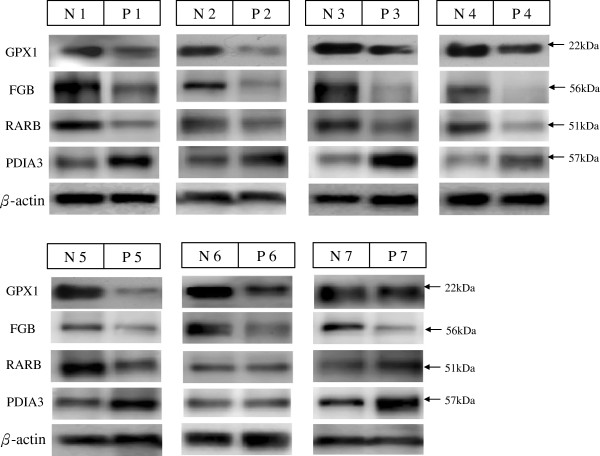
**Western blot analysis of human platelet proteins.** Validation of PDIA3, FGB (FIBB), GPX1, and RARB. Nos. N1–N7 indicate healthy control samples, and Nos. P1–P7 indicate patient samples.

However, because of the limited platelet amounts, only 7 patients and 7 controls (age- and sex-matched) were examined for protein validation by western blot analysis. Among the identified proteins, protein disulfide-isomerase A3 (PDIA3), fibrinogen beta chain (FIBB), glutathione peroxidase 1 (GPX-1), and retinoic acid receptor beta (RARB) were validated by western blotting. The results are shown in Figure 2. PDIA3 was up-regulated in the patients, whereas FGB, GPX-1, and RARB were down-regulated in the patients. Protein levels were quantified by densitometric analysis with the control being set at 1. Data presented as means ± SD of three independent experiments for each sample. The GPX1, FIBB and RARB were 0.66 ± 0.15, 0.45 ± 0.12 and 0.68 ± 0.17, respectively. The PDIA3 was 1.96 ± 0.39. The western blot analysis results of these 4 differentially expressed proteins were in agreement with the 2-D PAGE expression data.

**Figure 3:**
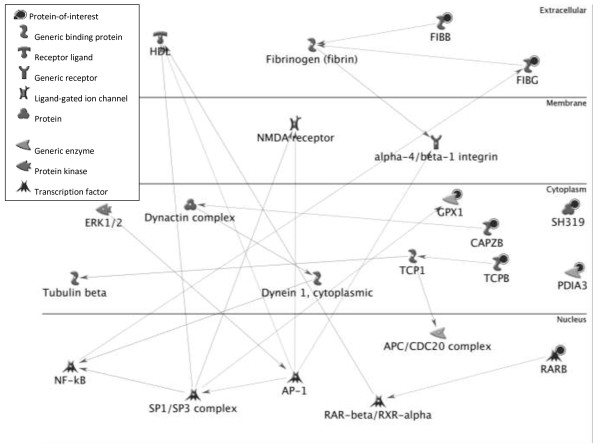
Networking construction of eight differentially expressed platelet proteins between healthy controls and patients with major depression using Metacore software from GeneGo Inc.

We used bioinformatic tools to classify these eight significantly differential display proteins. The identified proteins were mainly distributed in the cytoplasm, followed by nucleus and secreted form (Table 2). The protein SH319 and RARB located in the nucleus, but FIBB and FIBG were secreted according to UniProtKB/Swiss-Prot and GO databases. Primary molecular function was binding and these identified proteins abundantly bind to protein, nucleotides, nucleic acid, receptor, chaperone and actin (Table 2). The second molecular function was enzyme activity including retinoic acid receptor, transposing S-S bonds and glutathione peroxidase (Table 2). Further studies were performed to construct protein-protein interaction network and analyze correlation between disease and identified proteins by the Metacore from GeneGo Inc. Nodes with varied symbols stand for different types of proteins and lines with different colors between nodes indicate different protein-protein regulation modes. Moreover, we put these eight identified protein together and tried to assemble protein-protein correlationships (Figure 3). There are mainly two transcription factor, namely SP1 and AP-1 inclusive in the protein network. Other transcription factor includes ERK1/2, c-Jun, c-Myc, c-Fos, RelA, HSF1, Elk-1, STAT3, NF-κB, Oct-3/4 and SP3. The membrane receptors primarily are integrin family (alpha 2, alpha 4, beta1, alpha 4/beta 1, alpha 5/beta 1, alpha 5/beta 3), Plexin A1, Thrombomodulin, Tissue factor.

**Table 2:** Functional classification of differentially expressed platelet proteins between healthy control and patients with major depression by using BiNGO 2.44 software

**Component**	Protein symbols*
Cytoplasm	FIBB, FIBG, TCPB, GPX1, FIBG, RARB, GPX1, PDIA3
Membrane-bound vesicle	FIBG, PDIA3, FIBB
Stored secretory granule	FIBG, FIBB
Nucleus	SH319, RARB
Chaperone-containing T-complex	TCPB
Molecular function	
Protein binding	FIBB, FIBG, TCPB, GPX1, FIBG, RARB, GPX1, PDIA3
Cell surface binding	FIBG, FIBB
Retinoic acid receptor activity	RARB
Proline-rich region binding	SH319
Intramolecular oxidoreductase activity	PDIA3
Glutathione peroxidase activity	GPX1
Biological process	
Wound healing, response to metal ion	FIBG, FIBB, GPX1
Positive regulation of cell death	PDIA3, RARB, GPX1
Protein polymerization	FIBG, FIBB
Muscle cell differentiation	RARB, GPX1
Cell redox homeostasis	PDIA3, GPX1

*FIBB: fibrinogen beta chain, TCPB: T-complex protein 1 subunit beta, GPX1: glutathione peroxidase 1, FIBG: fibrinogen gamma chain, RARB: retinoic acid receptor beta, PDIA3: protein disulfide-isomerase A3.

The SH319 and PDIA3 seem to be excluded; however, both of them were indirectly related to SP1. The SH319 and SP1 activate a disintegrin and metalloprotease domain 17 (ADAM 17), as a tumor necrosis factor-alpha converting enzyme (TNF-alpha converting enzyme, TACE); binds mitotic arrest deficient 2 protein; and also involves in the activation of the Notch signaling pathway. The PDIA3 activate calreticulin (molecular calcium-binding chaperone) to hamper the binding of androgen receptor to its hormone-responsive DNA element and inhibit retinoic acid receptor transcriptional activities in vivo. The PDIA3 and SP1 activate MMP-9 to degrade collagen IV & V and fibronectin. The PDIA3 could also activate immune system through binding to MHC I for peptide antigen presentation and processing.

The eight identified proteins could be related to some disease developments according to Metacore database. Table 3 shows that skin and connective tissue diseases, breast neoplasms, pathologic processes, cardiovascular diseases, neuroectodermal tumors, neuroepithelial neoplasms, kidney diseases, wounds and injuries, and cerebrovascular disorders are associated with the identified proteins. The disease target proteins consist of FIBB, TCPB, GPX1, FIBG, RARB, and PDIA3. Among them, GPX1 and fibrinogen play ultimately crucial roles serving as biomarkers for multiple diseases.

**Table 3:** Correlationship between diseases and eight differentially expressed platelet proteins between healthy controls and patients with major depression using Metacore software from GeneGo Inc

**Disease**	Protein symbols*
Skin and connective tissue diseases	FIBB, TCPB, GPX1, FIBG, RARB
Breast Neoplasms	FIBB, TCPB, GPX1, FIBG, RARB
Pathologic processes	FIBB, GPX1, FIBG, RARB
Cardiovascular diseases	FIBB, GPX1, FIBG, RARB, PDIA3
Neuroectodermal tumors	FIBB, GPX1, FIBG, RARB, PDIA3
Neoplasms, neuroepithelial	GPX1, RARB, PDIA3
Glioma	GPX1, RARB, PDIA3
Kidney diseases	FIBB, GPX1, FIBG, RARB
Cerebrovascular disorders	FIBB, GPX1, FIBG
Wounds and injuries	FIBB, GPX1, FIBG

*FIBB: fibrinogen beta chain, TCPB: T-complex protein 1 subunit beta, GPX1: glutathione peroxidase 1, FIBG: fibrinogen gamma chain, RARB: retinoic acid receptor beta, PDIA3: protein disulfide-isomerase A3.

## Discussion

The most interesting finding in this study was higher PDIA3 expression and lower FGB, GPX-1, and RARB expression in patients with major depression than in healthy controls. Moreover, these proteins were validated by western blot.

PDIA3 is also known as a glucose-regulated protein; it is an isomerase enzyme with a molecular mass of 58 kDa and is essential for the formation of the final antigen conformation and exportation from the endoplasmic reticulum to the cell surface [41-43]. Data regarding the relationship between PDIA3 and major depression are scarce. Previous studies showed that the pharmacological actions of antidepressant drugs most affected the metabolism of two neurotransmitters: serotonin and noradrenaline. However, many aspects of antidepressant action are not yet understood. McHugh et al. [44] used a proteomic analysis in a neuronal cell culture model to identify new molecules relevant to antidepressant action [44]. After antidepressant exposure, they observed increased expression of PDIA3, sepiapterin reductase (SPR), and heat shock protein and decreased expression of creatine kinase, a T-cell receptor alpha chain, defensin-related cryptdin 5, and the intermediate filament protein glial fibrillary acidic protein. Therefore, PDIA3, SPR, and the other proteins identified might provide a link to the possible psychopathology of major depression.

With regard to FIBB, many studies have discussed the relationship between inflammation and depression. The blood markers of inflammation, including myeloperoxidase (MPO), interleukin-6, white blood cell count, C-reactive protein, tumor necrosis factor (TNF)-alpha, and fibrinogen, were noted [45,46]. Vaccarino et al. [45] showed a strong association of MPO and weaker association of other inflammatory biomarkers with major depression [45]. In addition, Danese et al. [46] showed that in patients with a history of childhood maltreatment, depression was associated with high levels of high-sensitivity C-reactive protein [46]. On the other hand, Markovitz et al. [47] found that depressed patients had greater platelet secretion than the comparison subjects in response to collagen; further, platelet secretion in response to collagen was significantly reduced after treatment with sertraline, an antidepressant [47]. Furthermore, several studies have attempted to establish relationships between fibrinogen, platelet, serotonin, BDNF, and major depression [8-10].

With regard to GPX1, there is some evidence that the activation of the immune-inflammatory process, increase in monoamine catabolism, and abnormalities in lipid compounds may cause overproduction of reactive oxygen species (ROS) and, in turn, antioxidative enzyme activities (AEAs) and lipid peroxidation (LP). These phenomena may subsequently be related to the pathophysiology of major depression. The antioxidant enzymes include superoxide dismutase (SOD), catalase, and glutathione peroxidase (GPX) and beta-adrenergic receptors [48,49]. Bilici et al. [48] showed that patients with major depression, especially melancholic patients, had higher AEA and LP levels than those of healthy controls. After treatment for 3 months with antidepressants, AEA and LP levels of the patients significantly decreased to normal levels [48]. Although there was a decrease in nitrite content and beta-adrenergic receptor binding in the patients with major depression as compared to that in the healthy controls, the activities of SOD, catalase, and GPX were not significantly altered in these patients [49]. However, major depression induced the increased risk for cardiovascular disorder (CVD) partially by significantly lower blood concentrations of major antioxidants and their enzyme activities as GPX [28,50]. Lang et al. [51] review molecular mechanisms of depression and search for perspectives on antidepressant treatment strategies [51].

With regard to RARB, several studies have recently shown that the molecular components required for retinoic acid signaling are expressed in the adult brain [52-54]. The overlap of brain areas implicated in retinoic acid function and stress and depression suggest that retinoids could play a role in affective disorders. Retinoids represent a family of compounds derived from vitamin A that perform a large number of functions, many via the vitamin A derivative retinoic acid. This signaling molecule binds to specific retinoic acid receptors in the brain, which, like the glucocorticoid and thyroid hormone receptors, are part of the nuclear receptor superfamily and regulate gene transcription [52]. An examination of vitamin-deprived animals revealed a progressive and ultimately profound impairment of hippocampal CA1 long-term potentiation. Importantly, these losses are fully reversible by dietary vitamin A replenishment in vivo or direct application of all trans-retinoic acids to acute hippocampal slices [53]. In a human study, Chen et al. [54] showed that retinoic acid receptors (RAR) might contribute to regulate the activity of corticotropin-releasing hormone (CRH) neurons in vivo, and the vulnerable characteristic of the critical proteins in the retinoic acid (RA) signaling pathways might provide novel targets for therapeutic strategies for depression [54]. In addition, Katsuki et al. [55] revealed that RAR stimulation protects midbrain dopaminergic neurons from inflammatory degeneration via BDNF-mediated signaling [55]. Further, cyclin-dependent kinase 5 (CDK5) might influence the formation of memory and mood by affecting differential effects of glucocorticoid on BDNF expression [56]. These results also indicate that neuroplasticity should be involved in the psychopathology of major depression. The limitation of this study was that a wash-out period of 1 week is very short. The antidepressants included paroxetine 20 /day (n = 3), Venlafaxine 150 mg/day (n = 2), Mirtazapine 60 mg/day (n = 2) and drug naïve (n = 3) in this study. We could not ascertain that there was has no effect on their results of the drug state of the patients.

## Conclusions

These findings suggest that the processes of inflammation/immunity, oxidative stress, and neurogenesis are involved in the psychopathology of major depression. These biomarkers infer the shared inflammatory and oxidative and nitrosative stress pathways that may contribute to major depression and result in cardiovascular disorders. To our knowledge, this is the first study to investigate the platelet proteins of major depressive patients by using 2D-DIGE, MALDI-TOF MS, and bioinformatic analyses. However, these data represent only preliminary results, and a large sample is needed to prove these results. In addition, the detailed functions of platelets in major depression need to be further studied in the future.

## Methods

### Patients with major depression and healthy controls

This study was conducted from December 2007 to November 2008 at Chang Gung Memorial Hospital (CGMH)–Kaohsiung Medical Center, Taiwan. The CGMH Ethics Committee formally issued an approval for this study. Patients suffering from major depression and healthy controls were selected according to the results of a Structured Clinical Interview for DSM-IV Axis I Disorders (SCID) [57]. Patients with other comorbid psychiatric disorders should be excluded, including bipolar disorder, schizophrenia, chronic fatigue syndrome and fibromyalgia. All the participants were free of liver, lung, renal, and metabolic diseases, and they did not take any medication for at least 1 week prior to entering this study.

### Separation of platelets from blood

Blood sample was collected into 1.5 eppendorf tubes; thereafter, 100 μl of ACD (acid-citrate-dextrose; Sigma C-3821), 300 μl of blood, and 660 μl of NaN_3_/PBS (0.05%) were centrifuged at 250 *g* for 10 min at 10°C. The supernatant was added to a new eppendorf tube and centrifuged at 1613 *g* for 10 min at 10°C. Next, the supernatant was discarded, and the platelets were washed in 1 ml of NaN_3_/PBS (0.05%). Thereafter, the platelets were centrifuged at 1613 *g* for 10 min at 10°C. After the supernatant was discarded, 200 μl of lysis buffer (8 M urea, 4% CHAPS, 30 mM Tris-Cl, pH 8.5) was added to the platelets, and they were stored at -80°C.

### 2D-DIGE analysis of the platelets

Total protein was quantified in 10 pooled control samples and 10 pooled patient samples using the Bio-Rad protein assay. The platelet proteins were then concentrated, and impurities removed using the 2D Clean Up Kit. The final precipitated pellets were redissolved in 60 μl of lysis buffer (8 M urea, 4% CHAPS, 30 mM Tris-Cl, pH 8.5), the total protein was quantified again, and the samples were stored on ice prior to subsequent Cy dye labeling. Each sample was labeled with 200 pmol (1 μl) of Cy dye per 50 μg of protein, incubated on ice for 30 min in the dark, and quenched with 1 μl of 10 mM lysine; each sample was then incubated on ice for 10 min in the dark, according to the manufacturer’s protocol. Ten control and 10 patient samples (50 μg of protein each) were labeled separately with either Cy3 or Cy5, and the internal standard (200 μg of protein comprising 25 μg from each of the 20 samples) was labeled with Cy2. One standard, control, and patient sample forming a set of Cy2-, Cy3-, and Cy5-labeled samples was combined for each of 10 gels and mixed with rehydration buffer (8 M urea, 1% CHAPS, 0.5% ASB14, 65 mM DTT, 0.5% v/v pharmalytes pH 3–10, 0.005% bromophenol blue) to a total volume of 250 μl per gel. The first dimension separation was performed on an IPGphor isoelectric focusing (IEF) unit (GE Healthcare) by applying combined samples to immobilized pH gradient (IPG) 13-cm pH 4–7 strips with rehydration at 30 V for 16 h followed by isoelectric focusing at 500 V for 1 h, 1000 V gradient for 1 h, and then 8000 V step for 32,000 V/h, for a total of 6 h and 30 V. Strips were each immediately equilibrated in a 5 ml solution of 6 M urea, 30% glycerol, 2% SDS, 75 mM Tris-Cl (pH 8.8), and 0.005% bromophenol blue for 15 min with 1% (w/v) DTT and subsequently for 15 min with 2.5% (w/v) iodoacetamide (IAA). A molecular weight marker (17–170 kDa) was added to one gel for reference. For the second dimension, strips were applied directly to 10% SDS-polyacrylamide gels, and 4 gels were run simultaneously on a SE600 (GE Healthcare) electrophoresis unit at 10°C at 0.35 W/gel for 16 h. Immediately after 2D-DIGE, the gels were scanned with a Typhoon Trio imager using an excitation/emission filter of 488/520 nm for Cy2, 532/580 nm for Cy3, and 633/670 nm for Cy5 to generate multiplexed DIGE image files. Statistical and quantitative analyses of the spot changes on the images were completed using DeCyder software (GE Healthcare).

### In-gel digestion and protein identification

Protein spots were excised from the gel, and the gel was washed 3 times with 200 μl H_2_O for 5 min by vortexing. The supernatant was discarded, and 200 μl of destaining agent (0.1 g K_3_Fe(CN)_6_ and 0.16 g Na_2_S_2_O_3_ in 10 ml dd H_2_O) were added by vortexing for 15 min. The supernatant was again discarded, and the gel was washed 3 times in 200 μl of washing solution I [50 mM NH_4_HCO_3_/100% ACN (3:2)] by vortexing for 15 min. The supernatant was discarded, and 200 μl of 100% ACN was added by vortexing for 10 min. Then, the supernatant was discarded, with a vacuum centrifuge for 10 min at room temperature (RT). After adding 6–8 μl of trypsin buffer (25 ng/μl trypsin, 25 mM NH_4_HCO) to cover the gels, the gels were digested in trypsin solution for 16 h at 37°C. The peptides obtained were extracted from the gel with extraction buffer (100% acetonitrile, 1% trifluoroacetic acid), concentrated with a vacuum centrifuge for 10 min at room temperature (RT), and rehydrated by the addition of 10 μl of deionized water.

Before their MS spectra and MS/MS fragment ion mass were determined with a Bruker MALDI-TOF/TOF Analyzer (Bruker Daltonics, Bremen, Germany), all product ions were submitted to a computer database search analysis with the Mascot MS/MS ion search (Matrix Science Inc., MA) by using the SwissProt database (all entries). The Bruker operation system, namely flexControl Version 3.3 (Build 85) and flexAnalysis software were used to create peak lists.

MALDI-TOF/TOF data were searched in-house MASCOT software (ver 2.2.04). The following parameters were used: enzyme, trypsin; variable modification, carbamidomethyl and oxidation; mass values, monoisotopic; peptide mass tolerance, ±100 ppm; peptide charge state, 1+; maximum missed cleavage, 1; significance threshold, *p* < 0.05. Peptide identification and protein assembly were performed in multiple stages. The protein identifications required detection of unique peptides and proteins with more than two spectral counts were selected for further analysis. The peptide mass data of each spot was submitted to the Swiss-Prot 100425 human species bioinformation stations using MASCOT search engines. Proteins identified with a higher MASCOT score in the bovine database than in the human database were considered as serum contamination and removed.

### Validation by western blotting and statistical analyses

The data of the platelet proteins were analyzed using DeCyder software (*P* < 0.05 in t-test; ratio of patient/normal >1.5 or <1.5) in 10 patient and 10 control samples. However, because of the limited amount of platelets, only 7 patients and 7 controls (age and sex matched) were examined for protein validation by western blot analysis. Protein levels were quantified by densitometric analysis with the healthy control being set at 1. Data presented as means ± SD of three independent experiments for each sample.

### Bioinformatic tools for protein searches and networking construction

The protein search programs used the following databases: NCBI (http://www.ncbi.nlm.nih.gov), and UniProtKB/Swiss-Prot (http://www.uniprot.org/). In addition, the functional protein association networks or protein interactions were searched by STRING database (http://string-db.org/). The combining pathway databases of BioCarta (http://www.biocarta.com/genes/index.asp), KEGG (http://www.genome.jp/kegg/pathway.html) and the PubMed literature (http://www.ncbi.nlm.nih.gov/pubmed/) were also used to search the correlated regulatory pathways in major depression and other related reactions. The MetaCore from GeneGo Inc. (Version 6.5) was applied in thoroughly compiling protein functional classification and interaction networks.

## Competing interests

The authors declare that they have no competing interests.

## Authors’ contributions

TLH and TYC conceived and designed the study. TLH diagnosed the patients and MLS collected the samples. MLS and TYC carried out laboratory work, collation of data, data analysis. TYC carried out bioinformatic data analysis and interpretation of mass spectrometry data. TLH and TYC prepared the first draft of the manuscript. All authors agreed with the final manuscript.

## Supplementary Material

Additional file 1: Table S1 Protein identification by PMF searching using Mascot software (ver 2.2.04). Click here for file
