# Supplementary material for: 2D-DIGE proteome analysis on the platelet proteins of patients with major depression
Source: Proteome Sci. 2014 Jan 3;12:1. doi: 10.1186/1477-5956-12-1 (PMC3898786; doi:10.1186/1477-5956-12-1)
Supplement: Additional file 1: Table S1 — Protein identification by PMF searching using Mascot software (ver 2.2.04). [file 1477-5956-12-1-S1.pdf]

Supplemental Table 1 Protein identification by PMF searching using Mascot software (ver 2.2.04)

Protein ID	Protein name	Accession No.	Mascot score	No. of matched/ total searched	Protein sequence Coverage (%)	Mr	Error (ppm)	Missed cleavage	Matched peptides	Unmatched peaks
TCPB	**T-complex protein 1 subunit beta**	P78371	114	16/56	41	1517.8628<br>819.4641<br>2288.2050<br>1419.8165<br>1291.7270<br>1781.9202<br>870.5335<br>2041.0271<br>1532.7975<br>2097.1061<br>1330.6584<br>2347.2407<br>1530.7194<br>2008.9927<br>1582.9122<br>2265.3472	-20.4<br>30.4<br>19.0<br>-3.82<br>0.043<br>-0.71<br>-8.23<br>-10.6<br>-24.6<br>-7.90<br>-0.96<br>18.8<br>15.0<br>-16.6<br>-2.55<br>13.1	0<br>0<br>0<br>1<br>0<br>2<br>0<br>2<br>2<br>0<br>0<br>0<br>0<br>0<br>0<br>1	R.LTSFIGAIAIGDLVK.S<br>K.VLVDMSR.V<br>R.VQDDEVGDGTTSVTVLAAELLR.E<br>K.KIHPQTIIAGWR.E<br>K.IHPQTIIAGWR.E<br>K.FRQDLMNIAGTTLSSK.L<br>K.LAVEAVLR.L<br>K.LGGSLADSYLDEGFLLDKK.I<br>K.ILIANTGMDTDKIK.I<br>R.LALVTGGEIASTFDHPELVK.L<br>R.GATQQILDEAER.S<br>R.MLPTIIADNAGYDSADLVAQLR.A<br>R.AAHSEGNTTAGLDMR.E<br>R.EGTIGDMAILGITESFQVK.R<br>R.QVLLSAAEAAEVILR.V<br>R.QVLLSAAEAAEVILRVDNIIK.A	823.1028, 825.1058, 839.0647, 839.5233, 841.0663, 845.0808, 847.0743, 855.0358, 856.5255, 861.0539, 864.4827, 865.0451, 870.9981, 877.0247, 907.9836, 971.6106, 1034.1022, 1039.5413, 1046.5386, 1050.0735, 1066.0516, 1068.5216, 1072.0489, 1082.0185, 1118.5228, 1130.5467, 1277.0547, 1296.6859, 1301.7106, 1323.7261, 1369.7401, 1554.7978, 1619.8230, 1758.9306, 1956.0751, 2093.0789, 2225.1284, 2233.1062, 2284.1828, 2366.1626
FIBG	**Fibrinogen gamma chain**	P02679	57	7/36	18	1491.7486<br>1513.7052<br>1682.9531<br>1117.5027<br>2233.0970<br>1034.5323<br>1545.8021	3.23<br>-15.2<br>-3.36<br>-22.0<br>33.2<br>5.59<br>-7.23	0<br>0<br>0<br>0<br>1<br>1<br>0	K.YEASILTHDSSIR.Y<br>R.YLQEIYNSNNQK.I<br>K.IHLISTQSAIPYALR.V<br>R.VELEDWNGR.T<br>R.VELEDWNGRTSTADYAMFK.V<br>K.VGPEADKYR.L<br>R.LTIGEGQQHHLGGAK.Q	908.0103, 945.5289, 1034.0803, 1046.5394, 1050.0908, 1066.0652, 1068.5215, 1072.0684, 1082.0384, 1100.3934, 1118.4630, 1132.4976, 1198.6889, 1277.0662, 1296.6832, 1363.6969, 1369.7232, 1482.7698, 1515.7365, 1619.8443, 1641.8155, 1758.9238, 1790.8658, 2093.0632, 2225.1120, 2247.0901, 2255.0510, 2284.1528, 2299.1299
SH319	**SH3 domain-containing protein 19**	Q5HYK7	63	8/19	11	2093.0975<br>1619.8183<br>839.5065<br>1641.8165<br>1046.5390<br>2225.0858<br>1369.7247<br>1363.6928	23.8<br>-5.69<br>39.5<br>-77.4<br>-60.9<br>-3.72<br>87.3<br>-32.3	1<br>0<br>0<br>1<br>1<br>1<br>0<br>0	-.MNIMNTEQSQNSIVSRIK.V<br>K.VFEGQTNIETSGLPK.K<br>R.AVQPAPTR.K<br>K.VPPERPPPPKLSATR.R<br>K.RGDVLVMLK.Q + Oxidation (M)<br>R.GDVLVMLKQTENNYLECQK.G<br>K.QTENNYLECQK.G<br>R.NQIGIFPANYVK.V	839.0724, 1030.1029, 1050.1022, 1066.0782, 1068.5368, 1082.0604, 1118.4912, 1277.1231, 1296.6864, 1758.9357, 2233.1328
CAPZB	**F-actin-**	P47756	70	8/30	26	1108.6777	27.4	1	R.RLPPQQIEK.N	1046.5664, 1050.0941, 1066.0720,

	**capping protein subunit beta**					952.5159<br>1518.7105<br>1696.8126<br>1568.7519<br>1534.8364<br>1337.6937<br>1171.6281	-31.8<br>14.0<br>-9.58<br>11.5<br>4.31<br>34.3<br>24.5	0<br>0<br>1<br>0<br>0<br>0<br>0	R.LPPQQIEK.N<br>K.YDPPLEDGAMPSAR.L<br>R.KLEVEANNAFDQYR.D<br>K.LEVEANNAFDQYR.D<br>K.LTSTVMLWLQTNK.S<br>K.SGSGTMNLGGSLTR.Q<br>R.STLNEIYFGK.T	1068.5363, 1072.0732, 1082.0433, 1193.6279, 1231.5983, 1277.1138, 1296.7533, 1369.7806, 1606.7264, 1619.8401, 1685.7644, 1758.8822, 2092.8093, 2210.7418, 2217.6719, 2224.7481, 2232.7198, 2283.7400, 2286.5673
RARB	**Retinoic acid receptor beta**	P10826	61	6/13	11	1329.6550<br>1296.7005<br>1046.5416<br>862.5382<br>1118.5324<br>1103.7243	16.6<br>91.2<br>-34.3<br>27.5<br>-96.4<br>74.8	0<br>1<br>1<br>1<br>1<br>1	R.VYKPCFVCQDK.S<br>K.NMIYTCHRDK.N + Oxidation (M)<br>K.NCVINKVTR.N<br>K.IVEFAKR.L<br>K.ILMKITDLR.S + Oxidation (M)<br>K.ITDLRSISAK.G	861.0773, 1015.6950, 1050.1158, 1051.6126, 1066.0961, 1068.5233, 1369.7604
PDIA3	**Protein disulfide-isomerase A3**	P30101	86	14/44	25	1191.6357<br>1084.5869<br>1236.5511<br>995.5623<br>1758.8410<br>1619.7985<br>877.5435<br>1172.5916<br>1188.5711<br>1359.6945<br>1515.7754<br>1664.7194<br>1341.7092<br>1370.7522	29.7<br>18.0<br>31.1<br>-0.97<br>-34.7<br>9.04<br>-40.6<br>43.7<br>30.0<br>26.9<br>10.8<br>-23.4<br>19.0<br>41.7	0<br>0<br>0<br>0<br>1<br>0<br>0<br>0<br>0<br>0<br>1<br>0<br>0<br>0	R.LAPEYEAAATR.L<br>K.YGVSGYPTLK.I<br>R.DGEEAGAYDGPR.T<br>K.QAGPASVPLR.T<br>K.QAGPASVPLRTEEEFK.K<br>K.DLLIAYYDVDYEK.N<br>K.LNFAVASR.K<br>K.FVMQEEFSR.D<br>K.FVMQEEFSR.D + Oxidation (M)<br>R.FLQDYFDGNLK.R<br>R.FLQDYFDGNLKR.Y<br>K.MDATANDVPSPYEVR.G<br>R.GFPTIYFSPANK.K<br>R.ELSDFISYLQR.E	825.0363, 839.0073, 845.0229, 847.0094, 854.9858, 856.4651, 861.0057, 864.4262, 864.9927, 867.0023, 870.4878, 876.9810, 882.9904, 892.9517, 907.8905, 1046.5684, 1050.0938, 1066.0753, 1068.5364, 1072.0782, 1277.1163, 1296.7485, 1363.7364, 1641.7950, 2210.6504, 2224.6699, 2232.6379, 2246.6283, 2283.6380, 3335.6486
FIBB	**Fibrinogen beta chain**	P02675	88	18/84	35	822.4093<br>1768.6757<br>844.4409<br>980.4436<br>836.3720<br>1308.6580<br>1691.7291<br>1535.7298<br>1820.6993<br>1275.6158<br>1239.5808<br>997.4466<br>1668.6792<br>1544.7014<br>886.3595<br>902.3446<br>1014.5636<br>1032.5928	-59.3<br>-63.0<br>-56.6<br>0.36<br>-69.5<br>30.7<br>-43.1<br>3.93<br>-60.5<br>43.7<br>50.9<br>19.9<br>-21.5<br>4.10<br>-31.7<br>-42.0<br>80.0<br>29.3	0<br>0<br>0<br>0<br>1<br>0<br>0<br>0<br>0<br>0<br>0<br>0<br>0<br>0<br>0<br>0<br>1<br>0	R.GHRPLDK.K<br>K.DNENVVNEYSSELEK.H<br>R.SILENLR.S<br>R.QDGSVDFGR.K<br>R.KWDPYK.Q<br>K.QGFGNVATNTDGK.N<br>R.MGPTELLIEMEDWK.G<br>K.AHYGGFTVQNEANK.Y<br>R.TMTIHNGMFFSTYDR.D<br>R.DNDGWLTSDPR.K<br>K.EDGGGWWYNR.C<br>R.CHAANPNGR.Y + Carboxymethyl (C)<br>R.YYWGGQYTWDMAK.H<br>K.HGTDDGVVWMNWK.G<br>K.GSWYSMR.K<br>K.GSWYSMR.K + Oxidation (M)<br>K.GSWYSMRK.M<br>K.IRPFFPQQ.-	825.0445, 827.4146, 839.0140, 845.0436, 854.9998, 861.0232, 864.4417, 866.4278, 868.3622, 870.5037, 877.0013, 898.3653, 904.4839, 908.3453, 926.4664, 948.4430, 960.4437, 963.4156, 986.5414, 996.4542, 1033.5890, 1046.5714, 1050.1158, 1054.5780, 1066.0922, 1070.5540, 1073.1095, 1076.5756, 1168.6713, 1180.6557, 1184.6693, 1221.5696, 1251.5841, 1255.5679, 1261.5508, 1271.5539, 1277.1430, 1287.5888, 1291.6357, 1296.7496, 1369.7641, 1384.6403, 1552.6820, 1557.7157, 1566.6911, 1574.6659, 1587.6888, 1619.8114, 1785.6800, 1807.6606, 1823.6700, 1849.8405, 1950.7951, 1966.7657,

										1991.7151, 2106.7391, 2126.7337, 2138.7083, 2148.6676, 2156.5602, 2160.5913, 2210.6565, 2232.6783, 2256.6157, 2283.6228, 2384.5766
GPX1	**Glutathione peroxidase 1**	P07203	79	8/50	45	1297.5854<br>2115.0447<br>1987.9951<br>1758.9020<br>1131.6103<br>1122.5064<br>1169.6787<br>1325.7719	1<br>0<br>11<br>16<br>11<br>-13<br>9<br>2	0<br>0<br>0<br>0<br>0<br>0<br>0<br>1	R.DYTQMNELQR.R<br>R.GLVVLGFPCNQFGHQENAK.N<br>K.YVRPGGGFEPNFMLFEK.C<br>K.CEVNGAGAHPLFAFLR.E<br>K.LITWSPVCR.N<br>R.NDVAWNFEK.F<br>K.FLVGPDGVPLR.R<br>K.FLVGPDGVPLRR.Y	823.0893, 825.0865, 839.0590, 841.0703, 845.0814, 847.0748, 855.0309, 856.5118, 861.0531, 861.5359, 864.4702, 865.0505, 867.0374, 870.5392, 870.9968, 877.0249, 892.9813, 908.0286, 1034.0968, 1046.5461, 1050.0792, 1066.0479, 1068.5322, 1072.0572, 1082.0180, 1118.5441, 1277.0790, 1296.6620, 1352.7619, 1364.7676, 1369.7526, 1496.8005, 1619.8449, 1653.9656, 1742.8472, 1790.9220, 1792.9239, 2093.0917, 2225.1268, 2233.1128, 2284.1838, 3337.9506
